# Toward a Real-World Technical Test Battery for Remote Microphone Systems Used with Hearing Prostheses

**DOI:** 10.1177/23312165231182518

**Published:** 2023-07-12

**Authors:** Michael A Stone, Melanie Lough, Keith Wilbraham, Helen Whiston, Harvey Dillon

**Affiliations:** 1 Manchester Centre for Audiology and Deafness, 5292 University of Manchester, Manchester, UK; 2 Hearing Device Research Centre, Hearing Health, National Institute of Health Research (NIHR) Biomedical Research Centre, Manchester, UK; 3 Department of Linguistics, Macquarie University, Sydney, Australia

**Keywords:** remote microphone, radio microphone, hearing aid

## Abstract

Remote microphones (RMs) enable clearer reception of speech than would be normally achievable when relying on the acoustic sound field at the listener's ear (Hawkins, J Sp Hear Disord 49, 409–418, 1984). They are used in a wide range of environments, with one example being for children in educational settings. The international standards defining the assessment methods of the technical performance of RMs rely on free-field (anechoic) delivery, a rarely met acoustic scenario. Although some work has been offered on more real-world testing (Husstedt et al., Int J Audiol 61, 34–45. 2022), the area remains under-investigated. The electroacoustic performance of five RMs in a low-reverberation room was compared in order to assess just the RM link, rather than measurements at the end of the signal chain, for example, speech intelligibility in human observers. It pilots physical- and electro-acoustic measures to characterize the performance of RMs. The measures are based on those found in the IEC 60118 standards relating to hearing aids, but modified for diffuse-field delivery, as well as adaptive signal processing. Speech intelligibility and quality are assessed by computer models. Noise bands were often processed into irrelevance by adaptive systems that could not be deactivated. Speech-related signals were more successful. The five RMs achieved similar levels of good predicted intelligibility, for each of two background noise levels. The main difference observed was in the transmission delay between microphone and ear. This ranged between 40 and 50 ms in two of the systems, on the upper edge of acceptability necessary for audio-visual synchrony.

## Introduction

Remote microphones (RMs) are a useful tool in conditions where speech communication is difficult. They enable the capture of audio close to the source, and delivery to a remote listener, by the use of electric wireless transmission. By doing so they mostly bypass the degrading effects of the acoustic conditions commonly present between source and listener. The delivery of a higher-fidelity signal at the remote location enables higher intelligibility, reduced listening effort, or both. This is especially useful to those whose hearing abilities are degraded (Maidment et al., 2018; Thibodeau, 2014).

RMs are commonly used in educational settings, where large room size, multiple competing audio sources, remote location of the listener from the teacher, and often poor acoustics, all contribute to the potential for degraded speech intelligibility at the listener's location (Anderson, 2004).

There are multiple methods of transmission of the RM signal to the listener (Dillon, 2012). When first developed, there were wires, but the technology progressed to use wireless connections such as magnetic transmission to telecoils, or radio signals where the analogue microphone signal was impressed on a radio carrier, such as by Frequency Modulation (FM). The ability to convert analogue signals into a digital data stream opened up a wide choice of data packaging techniques that use different (and often incompatible) radio carriers. Examples of such “streaming” techniques include WiFi as well as manufacturers’ proprietary systems. In practice, the data packaging technique is largely irrelevant, provided that it can reliably deliver good quality audio to the listener with only a short delay between source and listener.

The recent launch of a potentially less proprietary wireless digital data standard, Bluetooth^TM^ “Low Energy” (BT-LE, EHIMA, 2021), is of particular interest in classroom settings because it features a broadcast mode, “one [source] to many [receivers].” As a ubiquitous technology, Bluetooth entry costs are far lower for manufacturers than the use of proprietary systems, enabling a wider range of assistive products to be introduced (Smith & Davis, 2014).

In order to make comparisons between competing devices in many technologies, standardized testing methods have been developed. Of particular relevance to this report concerning RMs for hearing prostheses, are the IEC standards 60118-0 (IEC, 2015), and 60118-8 (IEC, 2005), which concern the testing of hearing aids (HAs). The IEC standards provide test batteries that assess the technical performance of HAs connected to an acoustic coupler in free-field conditions, either a test box (60118-0) or *in situ* (60118-8) in an echo-less room. An acoustic free-field represents an ideal acoustic environment since it has negligible reverberation.

A free-field acoustic environment is therefore unrelated to the environment in which a RM will be used. The signals described in the above standards form a good starting point for comparing sound capture and processing by an HA, but their presentation in the free-field would not address the competence of the technical solutions to problems that the RM is designed to overcome. There are many parallels between the IEC and ANSI family of standards, but the ANSI standards S3.47-2014 (ANSI, 2014) and S3.2-2020 (ANSI, 2020) are distinctive, being of relevance to the assessment of components remote from the HA as well as the measurement of speech intelligibility, respectively. Certain aspects of their design, such as the use of free-field-only signals, and the use of human observers for intelligibility tests, make them costly to implement for small-market devices.

A similar argument about the need for testing of RMs by using more achievable setups of equipment to measure real-world speech intelligibility has supported guidelines drawn up by European Union of Hearing Aid Acousticians (EUHA, 2017), based on evidence presented in Husstedt et al. (2022). The focus of that work is slightly different from ours, in that it aims to demonstrate the likely benefits to individuals, although still in a classroom environment. The work we report is driven more by the desire to be able to compare devices from different manufacturers by the use of a common objective test methodology but also being concerned with more than intelligibility. The measures are performed at an early stage in the signal chain, reducing or eliminating the additional complexities introduced by non-linear HAs and the influence of hearing impairment. The demonstration of benefits to individuals is seen as a subsequent step to this comparison process. A similar approach has been reported by Salehi et al. (2018), also testing in a more realistic acoustic scenario, but they only focused on metrics of intelligibility.

In order to compare the success, or otherwise, of delivery of good quality audio to the listener, our reported measurement technique employs real-body placement of the RM, sound delivery in room acoustics as well as computer models of speech intelligibility, and speech quality. The test scenario involves the simulation of a talker in the middle of a moderate sized, low-reverberation, room while wearing a microphone that is transmitting to a remote “listener.” The listener consists of a decoder connected to an HA. We consider the RM, its processing and transmission as one indissoluble system, since the current systems do not allow for the testing of signal quality within this chain. In order to assess the performance of just the wireless system, we are constrained to have to decode the radio signal in a manufacturer-specific receiver attached to, or integrated in, an HA. Since an HA can introduce distortions of its own, the HA was set to a low gain with linear processing, and all dynamic signal processing turned off. Any signal coloration introduced by the HA is therefore assumed to be small, when compared to those introduced by the wireless microphones. The inclusion of the HA is necessary since many systems require manufacturer-specific decoders, now often integrated into the HA. The HA is delivering audio into an IEC 60711 (ear simulator) coupler (IEC, undated) which itself contains a high-quality microphone. Recordings from this microphone can then be used to compare the loss of quality from the original acoustic signal received at the entry to the RM. “Listening boxes”, or “checkers”, where the decoded signal is available as an electrical output before reaching the HA, would make measurements easier to perform but are not commonly available across all manufacturers.

By concentrating just on the RM processing and transmission, and its decoding in a low-distortion receiver at the listener, we intended to avoid the pitfalls of the benefit being undone at the interface to the listener, such as would would be inappropriate mixing of the decoded RM signal with the acoustic signal received at the listeners’ HA microphones leading to near complete loss of the RM benefit (Hawkins, 1984).

In this pilot study, five wireless devices, all operating in the 2.4 GHz wireless band (where Bluetooth also is licensed) were compared. All five devices were in use in the UK education sector at the time of the study. Practically, one device was not a RM, but a remote receiver system (referenced later as “MUT E”). For this device the wireless output of a RM was converted on a receiver worn on the listener's body and re-transmitted on a 10.6 MHz radio carrier inductively coupled to the HA.

This pilot study provides a starting point to aid choice of a core set of electroacoustic measures, and methods, which could be used with RMs. The methods and measures are based on existing standards applicable in HA testing. The study explored the feasibility of, and issues that might arise with, more “real-world” testing. Such techniques should provide a uniform basis for comparison between device performance. Devices have, therefore, been anonymized. Some of the measures were not successful and so need addressing in any future work, if they are to be pursued further. Additional measures were taken on aspects of usability, but, for the sake of brevity of this report, will not be included here.

## Methods

### Equipment Setup

The torso of a KEMAR manikin was set up in the middle of a large “listening room” so as to represent the talker that the microphone system under test (MUT) was picking up. This room had dimensions 3.5 W×4.9 L×2.8 H m. The walls and ceiling were covered with sound-attenuating slabs, arranged in a semi-random pattern. Reverberation time (RT_60_) was uniformly flat across the 125 to 8,000 Hz range, around 120 ms. The physical volume was about 70% of the audiology room used by Husstedt et al. (2022), with a slightly shorter reverberation time (theirs was 140 ms).

**Figure 1.**
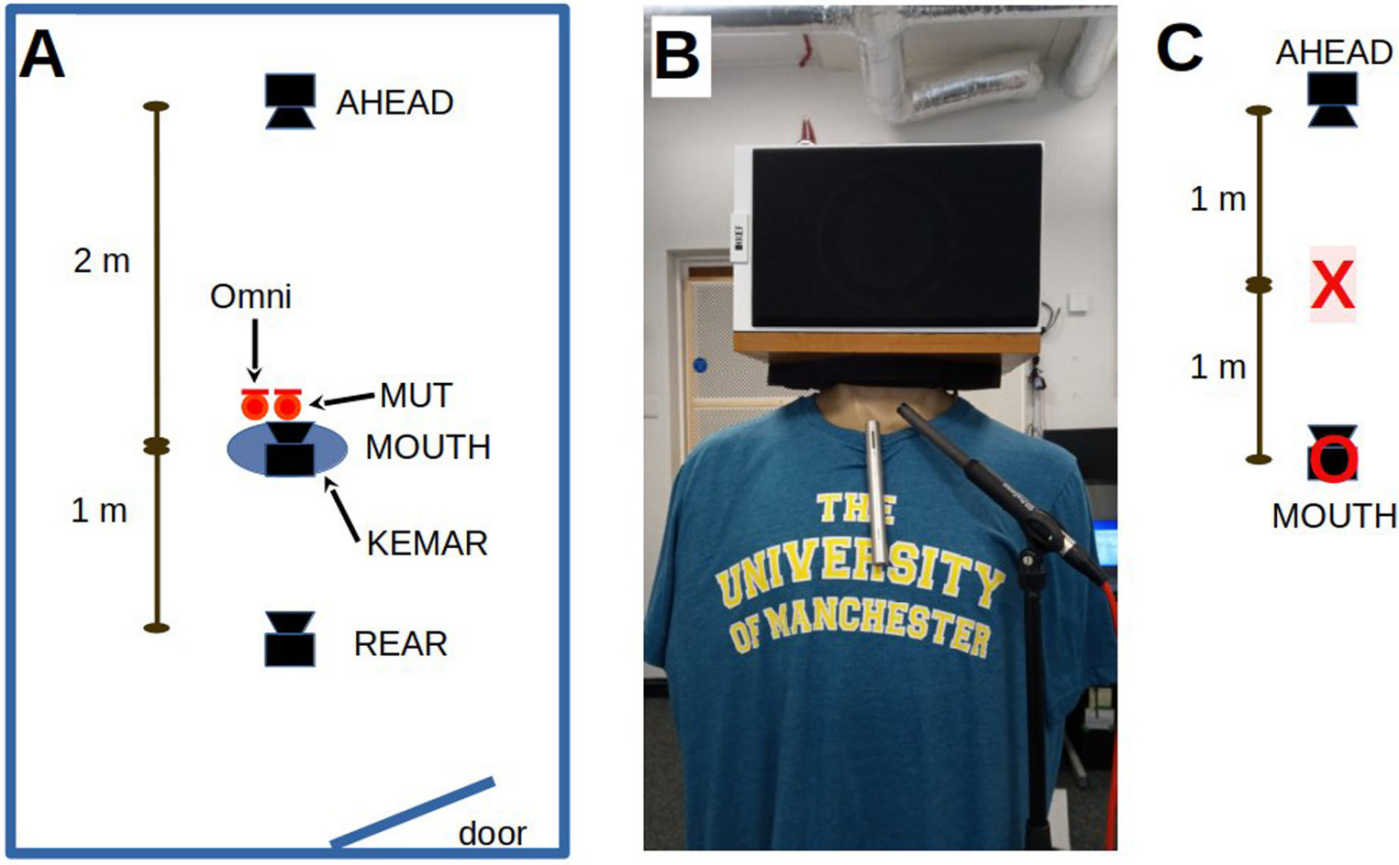
(A) The plan layout of the room, the orientation and distances between the three loudspeakers, and the positioning of the two microphones, the measurement microphone (Omni) and the MUT. (B) The positioning of an MUT attached to the neckline of KEMAR's shirt. The measurement microphone is positioned close to the MUT. (C) The reference measurement positions, **X** and **O** for the defining of speech and noise levels described below.

An adaptor plate was made that could carry a “bookshelf” loudspeaker, the KEF Q150 (30 × 28 × 18 cm (KEF Audio (UK) Ltd). This plate was attached to the neck of the torso, in place of the head. The shelf enabled the loudspeaker to be laid on its side, at approximately the same relative distance from the mouth to the (usually) high-chest-mounted RM of the system under test. The positioning of the apparatus around the KEMAR torso is shown in Figure 1, panel B. The Q150 has a “dual-concentric” transducer design: it has two drivers mounted on the same axis. One transducer, the 5.25″-diameter “woofer”, covers the frequency range up to about 2.5 kHz, while the other transducer, the 1″-diameter “tweeter”, covers the range above 2.5 kHz. The entire frequency range of the sound therefore comes from the same location, similar to a mouth. The Q150 has a very-near-flat on-axis acoustic response from below 100 Hz to above 10 kHz and so is well suited for both speech and music reproduction. Several studies report the relative frequency content of speech at different positions around, compared to in front of, the mouth, and these show generally an increasing drop-off of high-frequency content the further off-axis the measurement point is compared to the axis of the mouth (Dunn & Farnsworth, 1939; Flanagan, 1960; Monson et al., 2012). The loudspeaker provides sound radiated mostly in front of the cones, the diameter of the cone determining the frequency range at which the radiation becomes more directional. This directionality results in a loss of content off-axis from the loudspeaker similar to that observed around the mouth. Although “head and torso simulators” can come with “mouth simulators”, (a) the mouth simulator is mainly intended for use with microphones mounted close to the mouth, such as is common with telephones or headsets, (b) the loudspeaker in the mouth simulator is a compromise on size and quality, leading to distortion issues compared to the high-fidelity Q150, (c) they were beyond the budget of this study.

Two other Q150s were set up: one at 2 m in front of the manikin (AHEAD) and one at 1 m behind (REAR) of the manikin (see Figure 1, panel A). Although it is common to use loudspeakers at 1 m distance, reflections of the MOUTH signal from the AHEAD loudspeaker case could come back at an unacceptable level at the chest-worn MUT, confounding the measure there, hence the greater distance. The AHEAD and REAR loudspeakers were set up to produce a sound level of 65 dB(A) SPL in the middle of the manikin (with the manikin absent), position **O** in Figure 1, panel C. Loudspeakers AHEAD and REAR were intended for the delivery of interfering background noise, as well as a rough measure of relative directionality of the RM when attached to a torso. The manikin-mounted loudspeaker (MOUTH) produced 65 dB(A) SPL at a distance of 1 m in front of the loudspeaker, position **X** in Figure 1, panel C. The test signal for this measure was a speech-spectrum random noise, matched to the spectrum of the International Speech Test Signal (ISTS; Holube et al., 2010), a signal commonly found in hearing instrument test boxes (IEC, 2012). The horizontal axis of all three loudspeakers at their cone centers was aligned, 1.5 m off the floor.

The reference level of 65 dB(A) is equivalent to 68 dB SPL (unweighted), defined as “raised” in ANSI S3.5 (ANSI, 1997): slightly higher than is usually referenced in hearing instrument testing, but similar to what a teacher may produce in a classroom when “projecting” their voice.

### Playback and Recording Method

All of the measures proposed require pre-calculated test signals for presentation in a pre-calibrated acoustic environment. Playback and recording were performed by a single computer and a single soundcard. Playback required three channels; AHEAD, MOUTH, and REAR. Recording required two channels: (i) an omnidirectional measurement microphone adjacent to the MUT and (ii) the MUT itself.

The soundcard was a PreSonus Studio 26; a two-input, four-output-capable card (PreSonus Audio Electronics, LA, USA). An ART SLA-4 power amplifier (ART Proaudio, NY, USA) converted the soundcard outputs to drive the loudspeakers. The remote signal from the MUT was derived by decoding the signal in a relevant receiver HA set to the same modest gain across manufacturers, and no dynamic range compression (DRC), delivering via a 4-mm Libby horn inserted through an unvented foam earplug into the entrance of the 711 coupler in the Kemar head. Whereas some aids permitted a “Radio-Microphone-input only” mode, (muting the HA microphones), the HA paired with MUT A only permitted the HA microphone to be set at −12 dB relative to the input from the MUT. In order to reduce the leakage of the direct acoustic signal to the recorded output, either via the HA's own microphone or leakage around the foam earplug, the head carrying the coupler was situated in a cardboard box covered in acoustic wadding. This permitted recording of the HA signal in the same room as that in which the acoustic presentations were being made to the MUT. The microphone signal from the 711 coupler was amplified sufficiently to drive the soundcard.

### Hearing Aid Configurations

As a starting point, the HAs were programmed for a modest, flat-30 dB HL loss, using the NAL-NL2 prescription. In order to achieve the desired linear processing, the compression ratios were, however, all set to unity (linear) for levels between 50 and 80 dB SPL, and, using the widest bandwidth device as the reference, the gains were adjusted until they were within ±2 dB of each other from 250 to at least 8,000 Hz (except for the HA for MUT E, which could only achieve this balance up to 5,000 Hz). The test signal used was 45 s of the ISTS, presented at 65 dB to the aid sited in an Aurical HIT box (Natus Medical Inc., WI, USA). The recorded measure was the aided gain in a 2-cc coupler. The choice of audiogram produced a gain configuration (low gain, and further adjusted to be linear with level) such that the aids were unlikely to contribute much distortion to the measured results. Such distortions could arise from causes such as (1) too much electrical noise from the aid compared to the signal output, reduced by the use of some gain, as could be expected in realistic operation (2) DRC introducing non-linearities associated with replay level, (3) high output signal levels being distorted due to receiver limitations at the output of the aid, and (4) sustained high output levels causing temporary battery depletion. This latter manifests itself in test box measures as an apparent slow-acting DRC. It is an unintentional form of DRC since it arises due to a failure of the HA circuitry to operate consistently under conditions of a reduced battery voltage.

The drawback of using aided responses on which to perform measures is that most measures will be influenced by the relative across-frequency gain prescribed, which would vary according to the degree of hearing loss being compensated. Hence all reported measures are for signals that have been filtered to:

(1) Remove ultra-low frequency sounds, such as building vibrations (by forward and backward filtering with the same third-order high-pass elliptic filter with 0.1 dB passband ripple and 35 dB stop-band ripple. The filter was implemented in infinite-impulse response format. Its final response, after two passes, was −3 dB at 40 Hz).
(2a) Remove the aided gain response to produce an HA with flat 0-dB gain response referenced to the 2-cc coupler.
(2b) Reference the 2-cc response to the “eardrum” of the 60711 coupler (by use of a “2-cc to eardrum correction” response). This is the acoustic position at which we recorded the aid outputs to the received MUT signals.
(2c) Reference the 60711 eardrum response to the diffuse acoustic field by use of an inverse “diffuse-field to 60711 coupler response.”

For the measurement microphone recordings, only filtering stage (1) above was necessary. All filtering was performed with linear-phase filtering, and all filter delays were compensated, so as to preserve the time delay between the two-channel recordings.

The filtering process meant that all measures of the microphones are referenced to a recording position at the microphone of the MUT, as if it were sitting in a diffuse acoustic field. This is similar to that which would be found in a room with modest reverberation, similar to our listening room. Therefore, the HA gain is removed from the comparisons (although not any acoustic imperfections of the HA). In order to preserve the recording fidelity, the filtered signals were stored in 24-bit precision, still with the original sampling rate of 44.1 kHz.

### Parameter Measures

The proposed acoustic measures were based on the IEC 60118 standards used for the testing of HAs, specifically 60118-0 (IEC, 2015), but also incorporating the general background of *in-situ* testing as found in 60118-8 (2005). These measures comprised:

#### Frequency Response

This was measured across the range of 125–10,000 Hz using the ISTS, as well as a random noise with the same spectrum as the ISTS. Although the Speech Intelligibility Index (SII, ANSI, 1997) rates the frequency range of 400–4,500 Hz as especially important for speech intelligibility, there is evidence that bandwidth up to 10 kHz is, at least, of importance to children (Stelmachowicz et al., 2001, 2007).

#### Throughput Delay

Throughput delay is the time taken for the signal to travel from the RM microphone to the ear of the receiver. Low system delays are desirable so as to facilitate speech reading and the wider needs of audio-visual integration. McGrath and Summerfield (1985) recommended that delays should be kept below 40 ms so as not to degrade intelligibility for the most skilled lip readers. After accounting for HA delays, this should not be greater than around 35 ms in the MUT. In practice, acoustic leakage around, and via vents in, earmolds gives rise to the perception of the same signal but with two different delays; the preferred delay is lower than this, but that is for the sound source being close to the HA microphone (Stone & Moore, 2005). In an RM scenario, the acoustic path from the remote talker to the HA is longer, and so reduces the differential delay between the RM signal and the remote talker acoustic signal, leading, presumably, to acceptance of longer delays for that particular microphone pathway.

For this measure, the test signal was a bipolar click pulse with peak level of 87 dB SPL at 1 m. Each phase of the click was of 0.5-ms duration. Each click was separated from the adjacent one by 250 ms. Using digital audio editing software (CoolEdit 2000^TM^, Syntrillium, AZ, USA), the time interval around the onset of the measurement microphone response to the click was expanded. The interval between this and the appearance of the MUT response out of the recording noise floor was initially measured in the time waveform. In practice, many of the MUTs appeared to use transient suppression, so it was sometimes difficult to detect these in the time waveforms of the recorded outputs.

Onsets of narrowband noises were much easier to track in “spectrogram” representations of the signals. Identification of the onset, as well as any across-frequency variation in response was checked by switching to a spectrogram representation and looking for any deviation across time of the across-frequency view of the energy. In practice, none of the MUTs showed any across-frequency variation in delay. Due to temporal smearing in the spectrogram representation, it was only possible to measure to within 1-ms resolution. Although cross-correlation methods can obtain a finer measure, this degree of resolution was not necessary. Perceptual measures of the effects of audio-visual asynchrony only showed differences over much larger timescales (McGrath & Summerfield, 1985). Significant perceptual effects of HA processing delay also only manifest at intervals of multi-milliseconds (e.g., Stone & Moore, 2005).

#### Total Harmonic Distortion

Total harmonic distortion (THD) measures the amount of distortion generated by the system under test. A quality HA is unlikely to have distortion exceeding 2% since that will degrade the likely resulting clarity, and hence, intelligibility. IEC 60118-0 requires tests with pure tones at 500, 800, 1600, and 3200 Hz. These will work in a test box, but in a room, the acoustic “modes” of the room (despite its low reverberation) could lead to effects highly dependent on the position of the microphone. Hence narrow bands of noise are to be preferred.

We used 1/3^rd^-octave bands of low-noise noise (Pumplin, 1985) centered at 707, 1414, and 2828 Hz, at relative levels as they would be found in the ISTS when presented at a level of 68 dB SPL. The choice of low-noise statistics was originally made to permit higher replay levels from a digital file than is possible with noises with other statistics, for example, Gaussian. The frequency spans of the bands of noise were 630–800, 1260–1600, and 2520–3200 Hz, respectively, that is, with their upper edges close to the test frequencies required in 60118-0. The slightly different span of our test signals compared to those used for HAs was to ensure that any measured distortion components would be within a range of frequencies important for speech intelligibility. The levels of the signals were adjusted to be the same as the mean level of the same 1/3^rd^ octave band in the ISTS [−9.6, −14.2, and −18.4 dB, respectively, relative to the signal power in the full bandwidth]. These lower levels are necessary in case the processing in the microphone systems deems that the same signals presented at a higher level were not representative of speech and performed non-linear processing on them.

The low-noise property designed into the noises means that the signals exhibit a low crest factor, or peak-to-mean ratio and are therefore less likely to excite gain changes from any multi-band DRC within the MUT. Applying dynamic gain changes produces intermodulation, which would appear as a “distortion.” Any non-linear phase response in the loudspeaker, as well as reverberation, will degrade the low-noise property. For the MOUTH loudspeaker so close to the MUT, the effect of reverberation would be expected to be small, so the primary cause of degradation would be the non-linear phase response (if any) in the loudspeaker in the relevant frequency ranges.

#### Dynamic Range Compression

IEC 60118-0 requires measurement using tones at 2000 Hz, and, optionally, 707 Hz. Again, due to the presentation in a room, rather than a test box, 1/3^rd^-octave-wide bands of noise are the preferred signal type.

Two measures were required:

(1) Speed (attack and release times)

We used the 60118-0 testing method of providing a 35 dB step-change in level from −10 to +25 dB relative to the power levels in the long-term spectrum of the reference speech spectrum above and below 1414 Hz. The test signals were 1/3rd-octave bands of noise centered at 707 or 2000 Hz. The pulses had the same temporal pattern at both center frequencies:

- An initial 2-s duration “low” period (−10 dB) to provide a chance for adaptation.
- A 2-s duration “high” period (+25 dB) to test “attack”
- A 5-s duration low period (−10 dB) to provide a “release” period
- A repeat of (ii) and (iii).

The total duration of each test signal was therefore (2 +(2 + 5)×2) = 16 s. Theoretically, the compression ratio can also be determined from this test.

(2) Compression Ratio

Similar to the use in HA test boxes of a tone gradually increasing in level, we used 5-s duration steps of −10, −5, 0, +5, and +10 dB relative to the power measured in a 65 dB SPL ISTS signal, in both an ascending and descending sequence. Separately, three conditions were measured, one for a wideband noise, and two narrowband noises, either centered at 707 Hz or at 2000 Hz, with their levels referenced to the power in the 65 dB SPL ISTS signal measured below or above 1414 Hz, respectively. The use of noise is to avoid room modes, while the use of bursts longer in duration than found in a test box is to allow for any slow-acting level adaptation to have settled. The shape of the input–output relative growth in level should give the compression ratio.

#### An Alternative Method of Measuring Compression Ratio

The IEC 60118 family of standards does suggest a method for the assessment of HAs with non-linear processing. IEC 60118-15 (IEC, 2012) describes the use of the ISTS to characterize dynamic performance of HAs. Elberling and Hansen (1999) presented a method of measuring compression ratio by tracking the relative levels of short-time windows of signal, time-aligned, between the input and output signal, and computing the slope of the input-output function. This requires relatively long samples of the input signal in order to generate a large number of time windows. We therefore based our analyses on the four speech signals we had recorded for each MUT: (i) ISTS in quiet, (ii) male in quiet, (iii) female in babble, and (iv) male in babble.

After time-aligning between the master and the ear-level-received signal (this latter also being corrected for insertion gain from the HA and meatal effects) we calculated the logarithmic rms level in sequential windows, each of 125-ms duration, in both of the signals. The compression ratio can be derived from the slope of a regression performed on a scatter plot of the input levels against the output levels for each window.

#### Equivalent Input Noise

This is defined as the broadband equivalent noise at the input to the system when no signal is present. It is therefore independent of the subsequent amplification that may be provided by the HAs and is therefore comparable to real-world signal levels arriving at the microphone. Many electro-acoustic devices often incorporate low-level expansion, effectively turning off audio transmission when no input is present, so the Equivalent Input Noise (EIN) can appear to be very (i.e., unrealistically) low.

In order to ensure that the processing is still active, a low-level input signal is often necessary to “condition” the device so as to measure the system gain. The low-level signal is intended to be below the compression threshold of the subsequent system, so that the system gain is at maximum and can therefore be specified by the difference in level of the conditioning signal between input and output. From measuring the output signal level with no input applied, and subtracting the previously measured maximum HA gain, an estimate of the real-world EIN is obtained.

This measure is not entirely meaningful for at least two reasons:

1. The system gain will be different for the signals from the MUT and the HA's own internal microphone(s). These differences arise because of aspects of the MUTs’ adaptive processing, which applies level changes in the relative mix, as well as the absolute sensitivity of the MUT depending on additional measures of the signal-to-noise ratio (SNR) local to the listener.
2. As commonly defined, EIN is a broadband measure, and therefore does not reflect the perceptual interference that the noise may be having on localized frequency regions of the input signal.

We used a conditioning signal, presented at a low input level (50 dB SPL), so as to measure the SNR at the system output. Rather than just measuring overall SNR, we presented simultaneously four 0.28-octave noise bands each of 5-s duration, octave-spaced with center frequencies of 707, 1414, 2828, and 5656 Hz. There are therefore 0.72-octave gaps between the individual bands. The relative levels of the noise bands follow that of the powers in the same bandwidths of the reference speech spectrum. Spectrum analysis of the output signal then permits us to analyze the relative power between each signal band and the noise floor in the adjacent gaps. The SNR is defined as the mean signal density (dB/Hz) in the noise band compared to the mean signal density (again in dB/Hz) in narrow bands in the spectral gaps close to either edge of the noise band. This then gives us an idea of a localized SNR, specified at up to four points spread across the entire range of frequencies useful for speech intelligibility.

Rather than a single figure-of-merit for EIN, our four-band SNR method gives an idea of the likely ease of listening/disturbance due to the system background noise across the range of frequencies important for speech communication (ANSI, 1997).

#### Additional Measures, not Covered in IEC 60118-0

##### Predicted Speech Intelligibility and Quality

Performing intelligibility and quality assessments with real listeners is time-consuming and expensive. A range of software models have been developed to perform these functions. We used the recently updated version of the Hearing Aid Speech Performance Index (HASPI version 2; Kates & Arehart, 2021), and the Hearing Aid Speech Quality Index (HASQI version 2; Kates & Arehart, 2014), software meters which compare the recorded signal to the original signal to produce perceptually-based measures of an HA-processed speech signal. Another well-respected open-source software intelligibility meter commonly used in speech communication work is the ESTOI (Jensen & Taal, 2016), a model specifically designed to handle modulated maskers. All three of these models require a reference signal of the clean speech as well as a (near-) time-aligned version of the processed speech. Although their exact predictions may not track human-derived measures, their relative response is often reasonably accurate, and so can be used as the basis for ranking systems. As a baseline software measure, we also used the SII (ANSI, 1997). Although long-established, this measure is simplistic in its formulation in comparison to the models mentioned above, for example, it pays no attention to the modulation patterns in either the target or background noises and so is less consistent in its predictions (Rhebergen et al., 2006), and so proved to be less useful.

Using these models, we performed measures using single-talker female or male speech presented either in quiet or in a four-talker babble background noise. These two acoustic backgrounds represent two extremes of likely real-world use: ideal versus difficult. All four models produce a value that ranges from unity (excellent) to zero (abysmal). Translation of these metrics into exact intelligibility or quality depends on many factors, such as complexity of speech material, context, and especially in this application, the residual neural capabilities of the listener's hearing system. Hence we quote the metrics so as to enable the ranking of system performance.

The speech-in-noise measure was designed to simulate a classroom acoustic where there is multi-talker babble coming from ahead of the person wearing the microphone system. For this measure alone, the level from the AHEAD loudspeaker was reduced to 59 dB(A) measured at the position of the MOUTH loudspeaker (labeled **O** in Figure 1C), representing a + 6 dB SNR when referenced to the reference level of 65 dB SPL produced by the MOUTH loudspeaker as measured at the position labeled **X** in Figure 1C, 1 m away from the torso. As expected, and verified later, the SNR at the MUT was much higher because of the MOUTH speaker being closer to the MUT than the 1-m reference distance used to reference the SPL being produced by the REAR and MOUTH loudspeakers. For the speech-in-noise measure, we cannot report values for SII since that particular model needs an estimate of the babble signal alone at the microphone, as well as the (speech + babble) signal. Given that the presence of the MOUTH speech signal influenced the level of the babble spectrum due to the non-linear processing used in the MUT, then a babble-alone signal would not be the same as the babble component of the (speech + babble) recording. On the basis of requiring separate estimates of the speech and noise signals, the SII measure would likely be unrealistic and is therefore excluded from results for (speech + noise) measures reported below.

##### Directionality of the MUTs

Directionality defines the spatial selectivity of the MUT, that is, a measure of the relative response of the MUT to sound sources arriving from different directions. There are two concepts of directionality that can be invoked here:

(1) The relative response of the MUT to interfering noises coming from locations around the body of the talker.
(2) The relative response of the (high-chest mounted) MUT as the talker rotates their head relative to their torso.

Measurement (1) normally requires a very laborious procedure (unless automated): measuring the relative response at the microphone as a noise source moves around the MUT. This produces a “polar response” pattern. An example of a test methodology to do so is described in IEC 60118-8 (2005). Rather than a long procedure, we chose a shorter procedure.

Measurement (2) is necessary because, as the talker rotates their head relative to their chest, the microphone loses some of its sensitivity as the sound path between the two goes “off-axis” for both the mouth transmission and the microphone reception, relative to the axis used when in the straight-ahead position.

In order to cover these two concepts of directionality, we recorded the response of the MUT to 5-s burst of ISTS speech-spectrum-shaped noise arriving from either FRONT, REAR, or MOUTH. For the MOUTH measure, we started with the head pointed 45° to the left (as viewed from behind), then, during 6-s silent pauses, we moved the head to point to either 0° or 45° to the right, before a 5-s burst was re-played. Repeatability of adjustment was ensured by taping a straight edge to the middle of the MOUTH loudspeaker, and using that as a pointer to markers on the walls or to the middle of the FRONT loudspeaker at the relevant angles.

In these five measures, some of the observed differences will be due to the acoustic “shadowing” effect caused by the body of the talker, and some due to the test microphone response pattern. Again, this measure is more of a “real-world” indicator for comparison with other devices, rather than an abstract scientific measure.

## Results

The order of presentation of Results here parallels their description in the parameters subsection of the Methods section.

### Frequency Response

- *The influence of placing the microphone at the high-chest*

This measure does not rely on the processing in the MUT but indicates the properties of the acoustic signal from the MOUTH loudspeaker, as recorded at the measurement microphone, adjacent to the MUTs. The overall wideband level at the measurement microphone was 7.4 dB (unweighted) higher than the level that the MOUTH loudspeaker was producing at 1 m.

**Figure 2.**
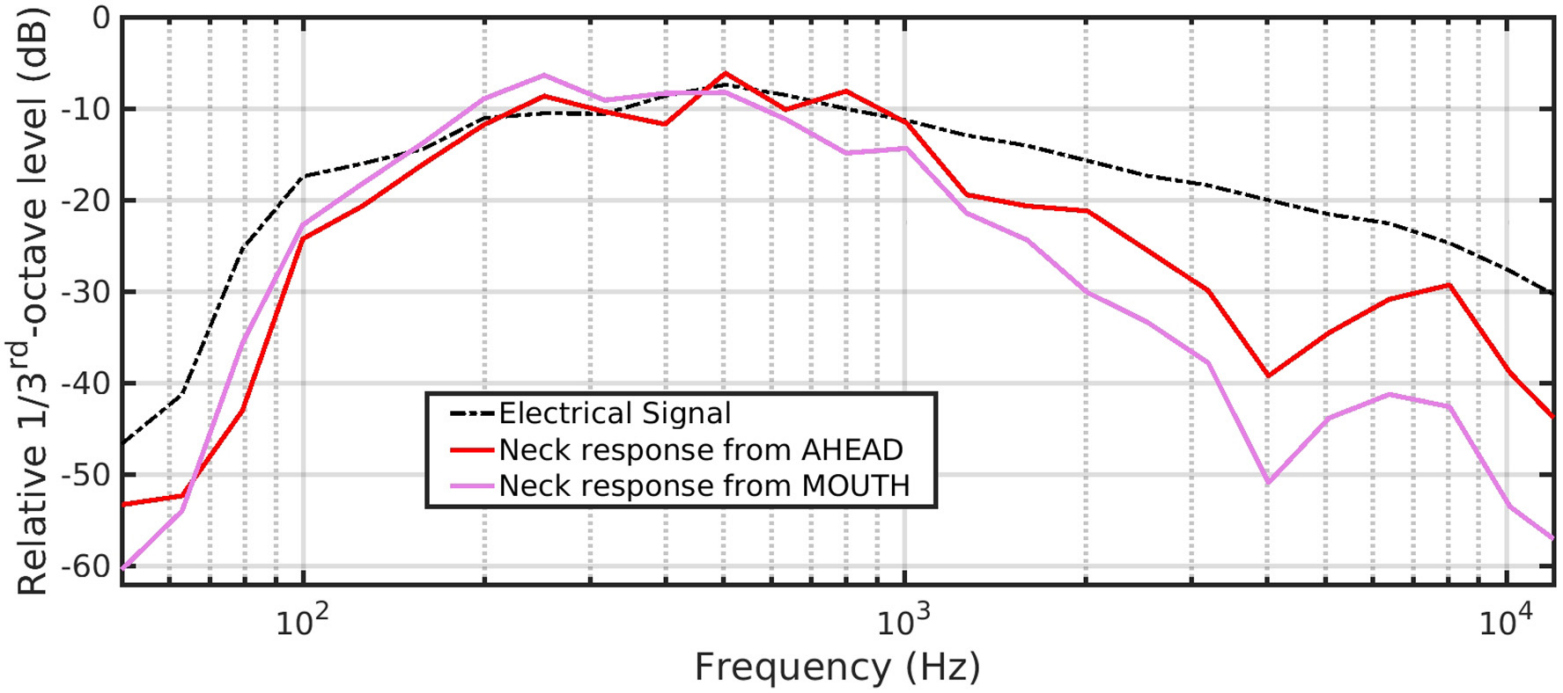
Comparison of relative 1/3^rd^-octave power between the electrical drive signal to the loudspeakers, and the response at the measurement microphone from either the AHEAD or the MOUTH loudspeaker.

Figure 2 shows the relative frequency responses of the calibration noise signals in the digital files, either as sent electrically to the loudspeaker (dashed black trace) or via the AHEAD (red trace) and FRONT (magenta trace) loudspeakers and recorded at the measurement microphone. The traces have been arbitrarily offset so that they align in level between 200 and 1000 Hz so as to demonstrate variation in response with frequency.

Compared to the electrical signal, the high-chest-recorded signals show a reduction at high frequencies (>1 kHz), varying between 5 and 15 dB, according to frequency. This is presumably largely due to the acoustic signal interacting with structures around the neck. It does mean that the tone quality of a microphone signal at the neck in response to the MOUTH signal will start off “muffled” compared to the original signal.

It is the muffling from a real mouth and head that the MUTs should be designed to overcome, otherwise it will result in lower audio quality and possibly reduced intelligibility. The muffling demonstrated here may be exaggerated compared to that from a real mouth because of the size of the loudspeaker defining the size of the associated support platform. Identifying a high-quality loudspeaker with a smaller (single-source) cone has proved difficult since it appears rare for such devices to come with technical details. A slightly smaller loudspeaker would permit reduction in the size of the support platform, or the use of a mouth simulator on a head-and-torso simulator, but with possible compromises in the signal bandwidth delivered. These changes together could be expected to make the simulated mouth to be closer in directionality than that achieved here.

(b) *Frequency responses of the systems in response to the ISTS*

**Figure 3.**
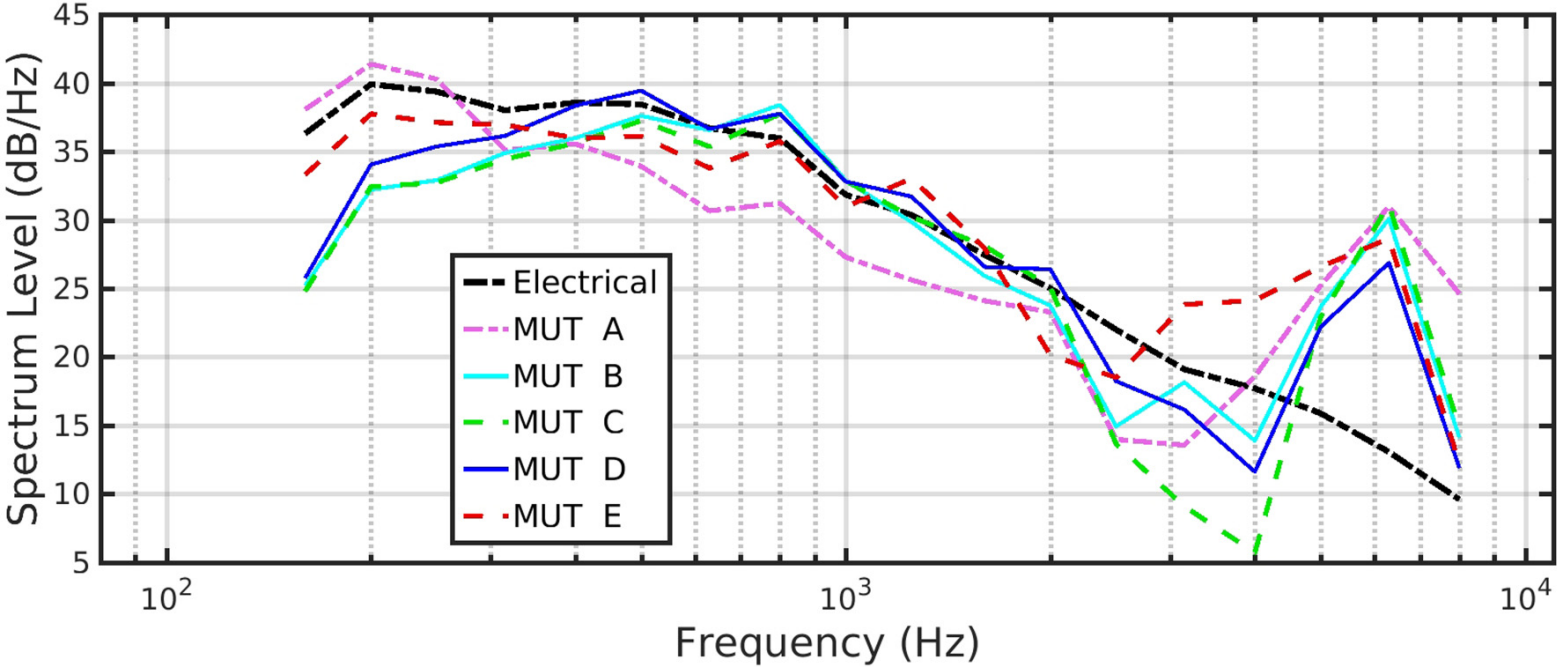
Comparison of the average system responses to the 60-s duration ISTS, normalized so that each has the same overall power.

This measure is very similar to the more recent method for measuring aid response in a test box (IEC, 2012). The average response to the 60-s duration of the ISTS is calculated. Figure 3 shows the average response for each of the systems. Notice that this is plotted in Spectrum Level, not 1/3^rd^-octave power, since it is the format necessary for input to one of the speech metrics, the SII (ANSI, 1997).

Note also that, for all recordings (i.e., except the electrical), there is a narrow peak around 6 kHz. Figure 2, red and magenta traces, also reported a smaller peak in this region recorded by the measurement microphone. We do not think this is due to the loudspeaker, but a possible interaction effect between the microphone position and the support for the MOUTH loudspeaker. Apart from this effect, these peaks could be not only due to pre-emphasis of this frequency region but also some system electrical noise increased in level by systems with high compression ratios. Since we cannot “break into” the signal chain between MUT and HA, we cannot identify the precise origin or balance of this noise relative to the speech.

For all systems, the response generally follows the reference (electrical response), except that MUT C does attenuate the 3–4 kHz region noticeably more than for the other systems. There is some low-frequency cut below 300 Hz in MUTs B, C, and D. Apart from these differences, there is generally no gross change expected to the “timbre” (tone quality) between these systems since, separate from a level offset that can be adjusted with a volume control, the responses are within 6 dB of each other (Caswell-Midwinter & Whitmer, 2019).

### Throughput Time Delay

This measures the delay between the acoustic signal entering the MUT and its being received at the microphone in the 711 coupler on Kemar's head. As such, it will also include any throughput delay of the HA. This is typically 8 ms maximum for a modern digital HA (Balling et al., 2020). It is reasonable to include the HA delay in these figures since a real user would have to use this processing path. HAs with delays shorter than 8 ms are available but tend to be used to correct mild-to-moderate losses. HAs with a sub 1-ms delay are currently rare, certainly for digital implementations of HA functions. Table 1 details the results.

**Table 1.** Comparison of Signal Delays from System Microphone Through to the Microphone in the 711 Coupler. These are delays that would be experienced in “real-world usage.” That is, including HA processing delay (typically up to 8 ms) but excluding the equalization filter used in preparation of recordings for analysis.

MUT	Delay (ms)
A	47
B	24
C	23
D	24
E	43

Three of the systems were all comfortably within the 40-ms limit suggested by McGrath and Summerfield (1985) as a conservative boundary. This boundary was based on an observation of a significant trend in declining lip-reading performance by the most able lip-readers over a range of asynchrony between 0 and 80 ms. Two MUTs, A and E, were above this, but, given the original derivation and definition of the boundary, not unacceptably so. We return to this in the Discussion section.

Since MUT E, the remote receiver, was being sourced by MUT B, then the true delay of MUT E alone was 19 ms. When working with multiple RM systems and HAs from different manufacturers, it is not uncommon to use bridging devices to ensure compatibility. Because these devices decode and re-code the digital audio multiple times, throughput delays can become quite long.

### Total Harmonic Distortion

Several of the systems used slow-acting DRC, possibly also coupled with noise reduction. So although we used a 23-s duration constant-level test signal, after about 7 s, aggressive reduction of level was appearing in many of the outputs. Therefore, we only measured the test signal for the first 7 s of presentation.

There was so little distortion in all of the systems that the system electrical noise dominated the measures. Once the measures were in the region spanning 5 kHz, the output was dominated by high-frequency electrical noise. It is because of these low distortion figures that we quote results in Table 2 in dB, rather than the conventional %. For comparison, measures less than −40 dB represent a < 1% THD, which is “probably” acceptable for HAs (Agnew, 1998).

**Table 2.** Comparison of Total Harmonic Distortion (in dB) Using 1/3^rd^-Octave Noises Around the Nominal Center Frequency, and Harmonic Numbers (H) Used in Calculation of THD. Those for 2828 Hz are entirely dominated by system noise, do not reflect distortion of the input signal, and so are italicised.

MUT	707 Hz (H2–H5)	1414 Hz (H2 and H3)	2828 Hz H2
A	< −40	< −37	*< −16*
B	< −29	< −43	*< −17*
C	< −32	< −40	*< −9*
D	< −32	< −34	*< −7*
E	< −35	< −44	*< −13*

MUT A therefore comes out as the least distorting of the systems, but only by a short lead.

Since the frequency region exceeding 4 kHz also contained system random noise, power from harmonics in this region has not been included, except for the measures of the 2828-Hz band signal since the second harmonic lies above 4 kHz. In fact, apart from some second harmonic distortion with systems B to E at 707 Hz, all other measures are dominated by system random noise, and so are comparatively meaningless in the sense that they are not primarily measuring distortion, especially the measures around 2828 Hz.

### Dynamic Range Compression

- *Attack and release times, including compression ratios using methods similar to 60118-0*

The attack and release times, as well as compression ratios, were virtually impossible to specify by the pulsed tones method required by IEC 60118-0. This was because of evidence of long-term (>10 s) adaptation to the test signals, leading to inconsistent performance between the first and second high-level portions, and their respective aftermaths.

None of the MUTs came with software to disable the non-linear processing aspects such as noise reduction, a software feature often used when testing HAs. Noise reduction is often performed by reducing the gain of channels in which the HA processing detects the statistical properties of (random) noise. This gain reduction is superimposed on any gain reduction due to the DRC, thereby confounding the measure. The procedures of IEC 60118-0 are therefore very hard to implement meaningfully in these MUTs.

(b) *Compression ratio shape measured by noise bursts stepped in level*

Although several of the MUTs showed waveform behavior typical of DRC in the first of the sequence of bursts stepped in level, subsequent bursts showed odd behavior implying some form of adaptive processing unrelated to the expected DRC. This sort of unclassifiable behavior was observed for all three test signals, wideband, low-frequency, or high-frequency. Results for this test will therefore not be reported further.

(c) *An alternative method of measuring compression ratio*

The scatterplots of the resulting pairs of input and output level for each 125-ms window showed a generally curvi-linear relationship between input and output over a wide dynamic range, with evidence of compression at the higher input levels, and more linear behavior at lower input levels. We therefore calculated the slope of a regression line over a range of levels spanning ±8 dB relative to the long-term rms of the input signal. This represented a deliberately narrower dynamic range of perceptual relevance than that normally assumed for speech (e.g., SII, ANSI, 1997), since (i) the possibility of compression limiting in the MUT might reduce signal peaks and bias the regression and (ii) the input signal dropping below the (unknown) compression threshold, might produce more linear behavior, again with a possible bias to the regression. Use of this dynamic range for the regression selected typically 50% of windows in the wideband signal, irrespective of presentation in quiet or noise.

The results are shown in Table 3 for both speech in quiet and speech in babble. MUT A exhibited a very high compression ratio, and MUT D exhibited a low compression ratio, almost linear in performance. Given the use of 125-ms analysis frames, this could also indicate the use of very slow dynamic range control, suggesting a low effective compression ratio (Braida et al., 1982; Stone & Moore, 1992) when measuring using short-duration signals.

**Table 3.** Comparison of Wideband Compression Ratios by Consideration of the Relative Levels of 125-ms Duration Windows Between Input and Output.

MUT	Wideband compression ratio			
	Speech in quiet		Speech in babble	
	ISTS	Male	Female	Male
A	4.90	8.14	7.77	10.50
B	1.85	2.01	1.71	1.70
C	1.61	1.79	1.51	1.37
D	1.24	1.31	1.06	1.14
E	2.13	2.77	1.93	2.17

### Equivalent Input Noise

The frequency-specific SNRs are shown in Table 4, referenced to the center frequency of the noise band around which the SNR was calculated. Although the measurement signal was of low input level, there were obvious signs in the broadband waveforms of reducing gain (possibly a combination of expansion and noise reduction) during the 5-s duration. Therefore, all measures were performed over durations where *most* of this adaptation had occurred (indicated in column 6 in Table 4). These SNR figures are encouraging since, even for a quiet input (50 dB SPL, equivalent to very quiet speech), there is still a dynamic range below the mean level that is not occupied by noise. For full access to speech articulation, the SII (ANSI, 1997) requires audibility of levels to −15 dB below the mean. The MUTs are therefore not applying a major limit on audibility before the signal reaches the HA.

**Table 4.** Frequency-Specific SNRs (in dB) for Each System.

MUT	Center frequency of noise bands (Hz)				Measurement duration of post-adaptation signal (sec)	Comments
	707	1414	2828	5656		
A	30	26	17	18	3.5	Stepped gain adaptation after first 1 s.
B	34	26	20	21	3.5	Gain adaptation more progressive through entire signal
C	32	27	14	22	3.5	ditto
D	20	28	17	21	3.5	ditto
E	34	30	21	8*	3.5	* beyond upper edge of HA matched gains

#### Objective Intelligibility and Quality Metrics of the Systems in Response to Speech Signals

The four software models mentioned earlier (SII, HASPI, HASQI, and ESTOI) were used to compare speech intelligibility and quality between the MUTs operating in both quiet, and with a multitalker babble “noise”.

- *Speech in quiet and noise (female and male)*

The MUT E recording level was generally much lower than for the other MUTs; in a real-world scenario, this could be adjusted by a change in volume control. Hence we have performed all of these measures for signals at the same input level to the models, a nominal 68 dB SPL. Although this approach is logical, it caused problems later when we considered the directionality of the MUTs, which will be addressed there.

Table 5 compares the model outputs for the final 45 s of the 60-s duration female (ISTS) and male speech signals in quiet (as required by IEC 60118-15). SII is a simple model, but it indicates that there is sufficient audibility for near-maximum intelligibility of speech. The HASPI, a more sophisticated intelligibility model than SII, agrees, with slightly larger (second decimal rather than third decimal place) differences between the systems. We list the sub-components of the HASQI measure, relating to the quality of the “non-linear” and “linear” distortions detected by the measure. Greater differences in measures are observed for this index than any of the others. This will be interpreted more after Table 6.

**Table 5.** Comparison of Speech Intelligibility and Quality Models for Both the (Female) 60-s ISTS and Male in Quiet. HASQI produces three measures for each run, sub-measures non-linear (Non-lin) and linear (Lin), and a composite (Comp) score from the sub-measures.

MUT	SII		HASPI		HASQI						ESTOI	
	ISTS	Male	ISTS	Male	ISTS			Male			ISTS	Male
					*Non-lin*	*Lin*	Comp	*Non-lin*	*Lin*	Comp		
A	0.993	0.991	0.999	1.000	*0*.*574*	*0*.*866*	0.497	*0*.*584*	*0*.*862*	0.503	0.858	0.841
B	0.995	0.993	0.987	0.999	*0*.*414*	*0*.*827*	0.342	*0*.*353*	*0*.*843*	0.298	0.825	0.753
C	0.995	0.993	0.979	0.992	*0*.*389*	*0*.*829*	0.323	*0*.*305*	*0*.*832*	0.254	0.816	0.734
D	0.997	0.994	0.990	0.998	*0*.*446*	*0*.*863*	0.385	*0*.*417*	*0*.*872*	0.363	0.888	0.824
E	0.995	0.989	0.992	1.000	*0*.*450*	*0*.*880*	0.396	*0*.*458*	*0*.*882*	0.404	0.808	0.745

**Table 6.** Comparison of Speech Intelligibility and Quality Models, as for Table 5, But for Each of 60-s Female and Male Continuous Speech in Babble.

MUT	HASPI		HASQI						ESTOI	
	Female	Male	Female			Male			Female	Male
			*Non-lin*	*Lin*	Comp	*Non-lin*	*Lin*	Comp		
A	0.969	1.000	*0*.*394*	*0*.*863*	0.340	*0*.*372*	*0*.*866*	0.322	0.759	0.714
B	0.957	0.999	*0*.*409*	*0*.*859*	0.352	*0*.*326*	*0*.*847*	0.276	0.804	0.728
C	0.867	0.990	*0*.*358*	*0*.*857*	0.307	*0*.*266*	*0*.*842*	0.225	0.784	0.695
D	0.873	0.996	*0*.*380*	*0*.*865*	0.329	*0*.*318*	*0*.*872*	0.277	0.825	0.748
E	0.967	1.000	*0*.*358*	*0*.*894*	0.321	*0*.*310*	*0*.*875*	0.272	0.706	0.631

Table 6 repeats three of the metrics as for Table 5, but this time separately using the final 45 s of the 60-s durations of female and male continuous speech in babble. The results for all three measures follow the same pattern as for the speech in quiet. For reasons detailed in the Methods section, the SII measures are not included because they would be unrealistic.

In both quiet, and the moderate level of noise used, the speech intelligibility metrics (HASPI and ESTOI) show the potential for high intelligibility. The main difference between the systems shows up in the sub-components of the quality metric, which primarily reflects variations in non-linear processing, such as DRC. The lower values from HASQI compared to those from the intelligibility metrics are not of concern. The HASQI metric ranges from zero to unity over a much wider range of SNR (typically 60 dB) than do the intelligibility metrics (typically 10 dB SNR). Figure 3 of Kates et al. (2018) suggests that, for a person with normal hearing, the numbers in Table 6 translate to an operating point with near-perfect intelligibility, but poorer quality due to operating at an equivalent SNR of slightly above 0 dB (assuming a random noise masker).

### Directionality of the MUTs

**Figure 4.**
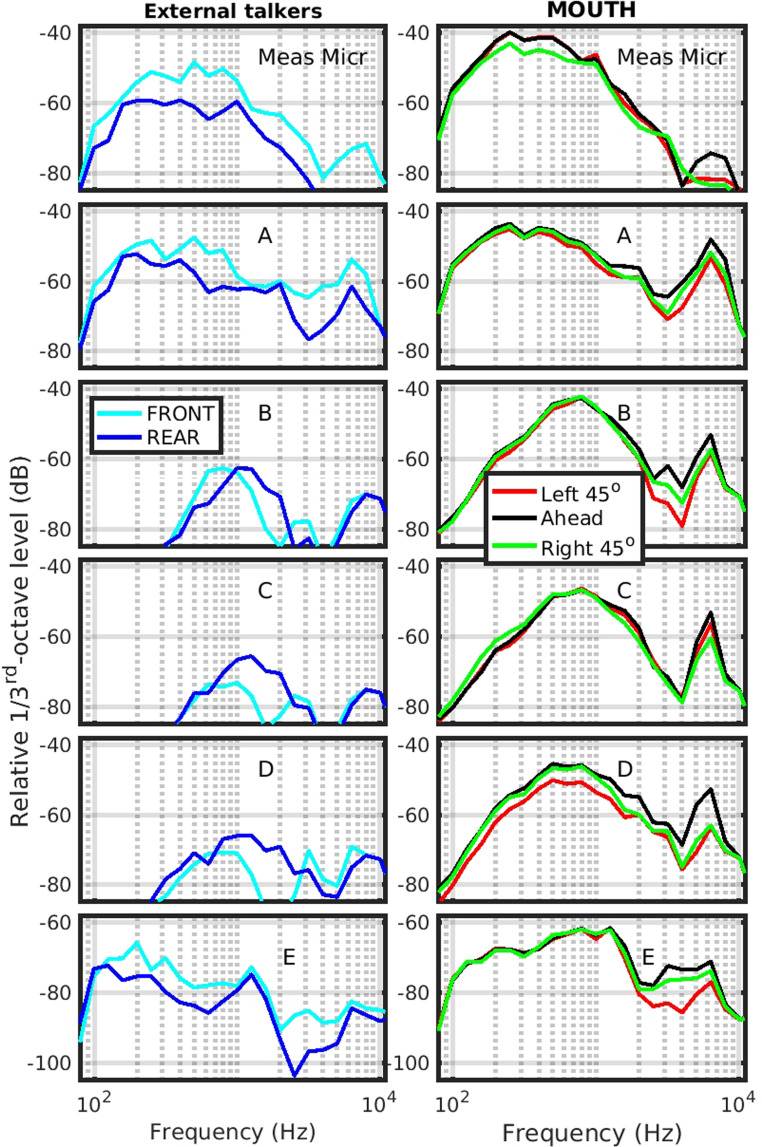
The relative signal level, in dB per 1/3^rd^-octave band for each MUT, including the measurement microphone (top panel of each column) in the directionality measures. The left-hand column shows the directionality for FRONT and REAR (external) sources, while the right-hand column shows the directionality for the MOUTH source at the three orientations relative to facing ahead. All traces are offset to maximize the plotting area, but the same offset is used between external talkers and MOUTH for each microphone.

With our set up, we therefore have two sets of measures for all five orientations, one set for the measurement microphone and one set for the MUT. We plot the signal levels received at each MUT for the different source locations, as shown in Figure 4. The levels are plotted as absolute signal level, in dB, measured in 1/3^rd^-octave bands for each MUT, as well as including the measurement microphone (top panel of each column). Because the signal level in the measurement microphone recording channel had a different sensitivity from the channel recordings of the MUTs as well as the MUT E signal being so low, then we cannot plot in absolute dB SPL. This is a minor detail, since we are primarily concerned with the pattern of behavior for each MUT between the left-hand column (external talkers) and right-hand column (the desired signal to be handled well by the MUT). We therefore plot the axes for all plots with the same span in dB, but with a different offset for each MUT.

Starting with the measurement microphone (“Meas Micr”, top panel), we see that the torso is in fact performing a general 10-dB blocking across all frequencies to the REAR talker. For the MOUTH signal, there is only a small difference as the MOUTH rotates, apart from above 4 kHz, where the left and right 45° signals are much lower than the AHEAD signal.

For the MUTs, especially for MUTs B, C, and D, we see a slightly higher mid-frequency (700–2200 Hz) response for the REAR source, than for the FRONT source, but overall, the response is much lower than the response for the MOUTH source. For all three systems, the AHEAD response is better than both of the 45° orientation responses, but there are only small differences (slightly larger for MUT D than for the other two), which is a desirable feature.

For the external talker signals, MUTs A and E receive the FRONT signal higher than the REAR signal, but with a lower sensitivity than for the MOUTH signals. Again, there is only a small change in sensitivity for the orientation of the MOUTH source (larger for the orientation of the MOUTH with MUT E). This latter finding is surprising since we used MUT B as the source microphone, so might have expected similar results as for that MUT. We can offer no explanation, other than that MUT B can switch between different directionality patterns automatically, such as may be useful when placed on a table, rather than body-worn. We did not manually set this directionality pattern, allowing it to auto-select.

It should be pointed out that these levels were calculated across the 5-s duration of the noise bursts used. Given that all MUTs, except for the measurement microphone, exhibited adaptive behavior, it is therefore likely that the responses are an average of a composite behavior. Since auditory perception can span timescales of hundreds of milliseconds (Moore, 2003), multi-second-duration averages are not always accurate predictors of perceptual effects. The caveat about composite measures not being the same as perceptual measures also applies to measurements in hearing instrument test boxes which are also based on medium-term averages (typically also using durations of seconds).

## Discussion

### Limitation and Opportunity

Although standardization of test methodologies and measures has enabled the production of clinically affordable test boxes, such as for HAs, the methods investigated by us, Salehi et al. (2018) and Husstedt et al. (2022), require the use of larger, and more specialist, facilities, and equipment. This will limit the widespread uptake of any measures developed from these bodies of work. Notwithstanding this, the methods presented here could be of utility before use in the clinic, namely to the commissioners of either health or education services when deciding on which devices to supply.

The availability of affordable multi-channel computer soundcards, as well as electrical “listeners” to decode the RM signal, combined with a software suite to perform analyses, has the possibility of making testing methods similar to those presented here available to a wider audience, albeit with some technical experience.

The work was commissioned by a set of end users who desire to see a tool for comparing devices independent from the range of measures provided by manufacturers in order to provide a “level playing field.” With the prospect of many systems being marketed based on common transmission standards, such as Bluetooth, there would appear to be a need for at least a first-level screening tool that assesses basic features of RMs so that suitability for a particular use can be assessed. For our commissioners, it was the use of RMs in a setting of pediatric education. A screening tool should be useful to reduce the need to run instances of larger, and more expensive user tests, such as comparisons of resulting intelligibility.

### Lessons Learnt for Future Studies

As a pilot study, we met several problems, some anticipated and some not.

#### Anticipated Problems

1. In trying to compare microphones we wished to intercept the remote signal as soon as it was decoded from wireless transmission. Although one manufacturer provided a “checker” for their transmitter, this was not the case with the other systems. We therefore used the output of a suitable HA that was programmed to have modest gain, and linear, rather than frequency-, or dynamic-range, compressed response. All other signal processing features of the HAs were de-activated (to the maximum degree possible) so that the output signal was a near-faithful reproduction of the decoded RM signal, as well as not constrained by the HA internal noise.
2. HA responses across different manufacturers should be near-identical, as verified in a hearing-instrument test box. Choosing a multi-channel programming model for each HA permitted a fair degree of frequency-specific fine tuning, so that this could be achieved.
3. Verifying the HA response. This was performed via an acoustic test box, and so required the HA microphone to be active. The aided-gain settings were copied between this program and a separate program on the same HA which would be set to “remote-microphone only” for its input so that the recorded HA response was solely from the RM. One then has to trust that the program-copy function works as expected, and that the HA manufacturer has not applied any extra processing (frequency shaping) to the RM signal compared to that of the microphone internal to the HA, and that the frequency response of the HA microphone to frontal signals in the test box is relatively flat when compared to the variation in insertion gain. These potential gain differences are not so critical in the Kates’ models which are, by default, intended to be used with different equalizations between the reference signal and the HA output signal. The Kates’ models, unlike ESTOI, incorporate an audibility threshold in their early stages. If the gain equalization is sufficient to cause the signal to drop below audibility then major changes in the resulting metrics can be expected.
4. Ensuring that the recorded HA response was solely from the RM. Most modern HAs are fitted with earmolds with various degrees of venting, from “open domes” to closed acrylic molds. The venting provides a bypass path, one of several ways that the acoustic field near the ear can affect the recording and reduce the effectiveness of the RM (Hawkins, 1984). Sound delivery from all HAs was via a 4-mm Libby horn inserted through a foam earplug into the entrance of the 711 coupler on the Kemar head. The head was placed in a radio-transparent cardboard box with wadding over the top to reduce the ingress of the acoustic field.
5. Our pilot study only reports measures in a low reverberation room with a modest volume (48 m^3^). Future development should investigate more realistic classroom volumes with longer reverberation times. Average volumes from large numbers of classrooms are around 198 m^3^ (41 measures in Canadian primary schools, Sato & Bradley, 2008) and 161 m^3^ (average of multiple rooms in 13 English secondary schools, Shield et al., 2015).

#### Unanticipated Problems

##### Working with HA Fitting Software from Multiple Manufacturers

This raised multiple issues:

- small changes in parameter settings caused non-linear processing features to become re-activated, requiring perpetual checking of settings between the two nominally identical programs in each HA, as alongside validation in the HA test box.
- “esoteric” behavior in fitting software where small changes to the software settings produced unpredictable changes in the HA response, requiring multiple iterations of fine-tuning and checking (such as gain changes in one frequency region drastically affecting one that was two octaves away, and large changes in gain and DRC being introduced when switching the tubing option).
- HAs from the same manufacturer, but with the same programming, had very different responses in the test box.

##### The Fast-Moving Nature of the Technology

Between the original definition of the workplan before the COVID-19 pandemic, and its post-pandemic execution, several changes occurred, such as two proposed devices becoming obsolescent, as well as integration of radio receivers into the HAs becoming more commonplace. Therefore parts of the assessment became difficult or irrelevant. Moving forward, a test suite needs to establish a base set of “essential” features in terms of functionality and perceptual utility, which supports the core reasons for the technology. The “non-essential” aspects of tests could still be useful in establishing a more rounded picture of the device.

##### Utility of Test Signals and Methods Chosen

Not all signals resulted in measures that discriminated between the different systems. A general problem, which has also been encountered in the testing of digital HAs, is that the MUTs incorporate proprietary signal processing algorithms that cannot be de-activated by the “fitting” software. Therefore all measures are prone to a bias because of adaptations within the MUTs, which occur over multiple different timescales. Any differences may only become apparent with more sophisticated (and longer-duration) test methods.

Of particular promise are software models of quality and intelligibility. We used the HASPI and HASQI because they are familiar to people within this field. Experience in Manchester has shown that these measures need a long sample of input, in excess of 20 s in order to obtain stable values. Given the observed adaptation times, it is suggested that around the 60 s detailed by IEC 60118-15 (IEC, 2012) is a suitable duration. We introduced the use of one other software model, the ESTOI, which is encountered more in the telecommunications field, but has an overlap with the radio communication aspect of the devices. All three software models require a copy of the original signal as well as the processed signal, so are more of a laboratory, than a “field”, measure. “Blind” software models, which only need a copy of the output signal, do exist, but they are not usually open source, so would require (expensive) licensing which is probably not justified/supportable by the size of this market at this stage.

##### Specifying the Throughput Delay

Correspondence by the first author with the European Hearing Instruments Manufacturers’ Association (EHIMA, 2022) revealed that low system delay has been a consideration in the design of the recently specified (early 2020) Bluetooth Low Energy standard (BT-LE), which contains the ability to broadcast from one microphone to many receivers, essential for a classroom-usage scenario. BT-LE, where data are streamed across radio waves to the receiver, is likely to become a common component of RM technology. Confidentiality agreements surrounding BT-LE forbid the detailing of the delays involved. This streaming introduces delays much longer than the more historic method of an audio signal directly changing the characteristics of a radio wave (e.g., the “FM” systems). Apart from the BT-LE delay, there will also be a delay introduced by the signal processing implemented by the RM manufacturer as they attempt to reduce background noise from the microphone signal. This latter component of delay is also company confidential.

An additional contribution to throughput delay is the possible use of multiple coders and decoders in order to achieve inter-operability between systems from different manufacturers. Each code/decode stage introduces additional delay so should be used sparingly. Although offered by at least one manufacturer, it is welcome that their literature does also advise against the regular use of this sort of solution.

For this report, we were not concerned with what exactly contributed to the delay. The nearest applicable evidence that can be used to define an “acceptable” delay is from McGrath and Summerfield (1985), on the basis of which we suggest adopting their precautionary approach which recommended that delays should not be much greater than 40 ms. The principle of an RM is to enable better access to speech cues which otherwise would get degraded in background noise and reverberation. It is not unreasonable for this principle to be extended to include preserving the perceptual link between visual and audio speech cues. We would therefore like to see the system delays routinely specified in data sheets.

## Conclusion

A pilot study using test methods similar to those already in use in the HA field (IEC 60118 and ANSI S3 families), as well as some adapted measures, has contrasted the technical performance of five different RM systems when used in a simulated “(near-) real-world” and “real-body” scenario rather than the “free-field” preferred by these standards. The RM systems exhibit sophisticated automatic digital signal processing which cannot be deactivated by the user. With the necessity to test the systems in a room rather than a test box, we were constrained as to which test signals were suitable: tone signals were best avoided. Other compromises were necessary, such as testing the systems via the acoustic output of a (presumed) low-distortion HA due to the absence of direct electrical output. This constraint does mean that some basic checks are performed on the HA to ensure that it is not the limiting factor in the signal chain.

The measures showed that the five systems compared were broadly similar in their performance. The main area where differences were exhibited were:

- Despite the difficulties of characterizing the DRC systems, further, meaningful, differences were observed in the software models of intelligibility and quality, which are broadly transferable into likely acceptability from a technical performance angle.
- Three systems, when used on their own, showed the best directionality in terms of ability to reject sounds arriving from away from the talker while also exhibiting only a small variability in output with moderate “head” rotation of the talker.
- The system delay was on the edge of acceptable for two of the systems, but well within acceptable for the other three systems. We urge manufacturers to report their system delays in their data sheets.

This report should be seen as an opening in a drive to standardize test methodologies across multiple technology types used in RM systems. Future work should especially consider (a) the use of test signals that are more speech-like in their spectro-temporal properties, so as to represent more real-world usage, and reduce the possible activation of signal-specific processing, such as (random) noise reduction (b) characterizing the boundaries of acceptable system delays in a pediatric population with hearing impairment.
